# Encapsulation of a *N*-Alkylamide-Enriched Fraction from *Acmella oleracea* and Its Efficacy Against *Tuta absoluta*, the Invasive Key Tomato Pest

**DOI:** 10.3390/insects17050455

**Published:** 2026-04-26

**Authors:** Simona Tortorici, Roya Namaki-Khameneh, Milko Sinacori, Eleonora Spinozzi, Filippo Maggi, Giada Trebaiocchi, Riccardo Petrelli, Diego Romano Perinelli, Thomas Giordano, Ernesto Ragusa, Luigi Botta, Haralabos Tsolakis, Gabriella Lo Verde, Roberto Rizzo

**Affiliations:** 1 CREA—Research Centre for Plant Protection and Certification, Viale Michelangelo, 1542, 90145 Palermo, Italy; simona.tortorici@crea.gov.it; 2 Department of Agricultural, Food and Forest Sciences (SAAF), University of Palermo, Viale delle Scienze, Ed. 5, 90128 Palermo, Italy; roya.namakikhameneh@unipa.it (R.N.-K.); milko.sinacori@unipa.it (M.S.); thomas.giordano@unipa.it (T.G.); ernesto.ragusa@unipa.it (E.R.); haralabos.tsolakis@unipa.it (H.T.); 3 ChIP—Chemistry Interdisciplinary Project Research Center, School of Pharmacy, University of Camerino, 62032 Camerino, Italy; eleonora.spinozzi@unicam.it (E.S.); filippo.maggi@unicam.it (F.M.); giada.trebaiocchi@unicam.it (G.T.); riccardo.petrelli@unicam.it (R.P.); diego.perinelli@unicam.it (D.R.P.); 4 Department of Engineering, University of Palermo, Viale delle Scienze Ed. 8, 90128 Palermo, Italy; luigi.botta@unipa.it

**Keywords:** South American tomato pinworm, botanical insecticide nanoemulsion, eco-friendly insecticide, phytotoxicity–ovicidal–larvicidal activity, ovideterrence

## Abstract

In the framework of integrated pest management, plant-based insecticides represent a promising tool for the control of insect pests. Indeed, *N*-alkylamides extracted from *Acmella oleracea* (L.) RK Jansen (Asteraceae) have been recently studied for their insecticidal properties. The encapsulation of these substances into stable formulations, like nanoemulsions (NEs), could boost their efficacy and stability. Herein, a *N*-alkylamide-enriched fraction (AEF) encapsulated into a stable NE was tested against *Tuta absoluta* (Meyrick) (Lepidoptera: Gelechiidae), a key tomato pest, able to develop resistance towards chemical insecticides. *Acmella oleracea* was reported to be effective against many target species, but this is the first time that this extract was tested against *T. absoluta* in terms of toxicity against eggs, ingestion toxicity on larvae and repellence on adults. The AEF, containing 42.8% of spilanthol, was prepared by combining two eco-friendly techniques, namely supercritical CO_2_ extraction and wiped-film short path molecular distillation, and then encapsulated into a stable NE. Preliminary tests on the phytotoxicity of the AEF-NEs at 0.25 and 0.5% (*w*/*w*) a.i., compared with a control NE solution (i.e., the AEF-free NE) and a negative control (distilled water), showed a negative effect on tomato plants at the highest concentration. On this basis, three concentrations (0.06, 0.125, and 0.25% a.i.) were evaluated against eggs (topical toxicity), larvae of 2nd instar (ingestion and topical toxicity), and adults (ovideterrence) of *T. absoluta*. The results showed that all adopted AEF-NE concentrations caused a significant inhibition in egg hatching (>20%). The larval survival, at the end of the evaluation (72 h), in ingestion toxicity tests were significantly different in the AEF-NEs at 0.06, 0.12, and 0.25% (56.7, 33.3 and 26.7%, respectively) compared with control NE and distilled water (100% both). Similar results were obtained in the adult emergence in ingestion toxicity comparing AEF-NEs at 0.06, 0.12, and 0.25% (64.7, 50.0 and 75.0%, respectively) with control NE and distilled water (100% both). Finally, a significant ovideterrent effect was shown by the concentrations 0.125 and 0.25% of the AEF-NEs (% of egg laid: 7.5 and 27.4% respectively), compared with distilled water. Overall, the AEF-NE tested showed promising and encouraging effectiveness as ovicidal and larvicidal against *T. absoluta*. This supports its potential use as an effective alternative to synthetic products for the control of this important pest.

## Simple Summary

The South American tomato pinworm *Tuta absoluta* is the key pest of tomato crops in almost all regions where tomato is cultivated. In recent years, control methods with lower environmental impact, as alternatives to chemical treatments, have increasingly been adopted. Among them the use of plant-based insecticides for the control of arthropods represents a potential control strategy. In this framework, the plant extract from *Acmella oleracea* with a *N*-alkylamide-enriched fraction (AEF) seems to be a successful tool in integrated pest management. This product was prepared by combining two eco-friendly techniques, namely supercritical CO_2_ extraction and wiped-film short path molecular distillation, and encapsulated into stable nanoemulsions (NEs) at different concentrations. After assessing the phytotoxicity on tomato plants, the AEF-NEs were tested against eggs, larvae and adults of *T. absoluta* in laboratory bioassays. The results showed that AEF-NEs inhibited egg hatching and reduced larval survival and adult emergence. Moreover, a significant ovideterrent effect was shown comparing different concentrations of AEF-NE with distilled water. This study indicates that the AEF-NEs could represent a promising alternative to synthetic pesticides against *T. absoluta* to reduce the insecticide resistance in the pest populations.

## 1. Introduction

Among the most important vegetable crops world-wide, tomato (*Solanum lycopersicum* L., Solanaceae) is cultivated on more than 5 million hectares, with a total production over 192 million tons [1]. Italy is one of the most relevant European producers, with 38% of the total production [1]. Tomato cultivation is constantly threatened by numerous arthropods, among which *Tuta absoluta* (Meyrick) (Lepidoptera: Gelechiidae) is one of the most important pests [2,3]. The insect, native to South America, is currently widespread as the key pest in almost all regions where tomato is cultivated. *Tuta absoluta* mainly attacks leaves and fruits of tomato plants, potentially causing yield losses up to 80–100% [4,5]. Moreover, it has been demonstrated that the insect is able to transmit tomato brown rugose fruit virus [6]. The management of *T. absoluta* relies on a variety of strategies, encompassing both preventive and curative approaches. Chemical insecticides remain the main method for controlling this pest [7,8]. However, alternative methods with lower environmental impact are increasingly being adopted. These include biopesticides, parasitoids and predators, pheromone and light traps, insect-proof nets, removal of infested plant parts, and solarization [5,9,10,11]. In recent years, new and environmentally sustainable sources of insecticides have been investigated, including plant essential oils/extracts or pure compounds [12,13,14,15,16,17,18,19,20,21].

However, while many studies addressed the insecticidal activity of essential oils/extracts from a large number of plant species, their chemical instability and scarce aqueous solubility limit their industrial production and use in the field [22]. For these reasons, in recent years, the use of nanocarriers have been also evaluated, to deliver the active compounds in a controlled and targeted manner, improving their efficacy while minimizing required dosages and possible environmental contamination [23,24,25,26]. Among them, the formulation of nanoemulsions (Nes) is one of the most investigated methods to encapsulate the plant derived compounds, in order to enhance their dispersion, stability, and bioavailability [27,28,29,30,31]. The effectiveness of NEs encapsulating plant extracts has been evaluated against several insects species of agriculture and medical interest, like aphids [25,32,33], lepidoptera [34,35,36], mosquitoes [37,38,39], stored products beetles [40,41], and mealybugs [42].

In the present study, we evaluated the activity of *Acmella oleracea* (L.) R.K. Jansen (Asteraceae), a widely cultivated species, which is a South American native herb, commonly referred to as jambù, and currently recognized for its applications in the food, cosmetic, and pharmaceutical sectors worldwide [39,43,44,45,46,47]. The extract from *A. oleracea* inflorescences is characterized by bioactive *N*-alkylamides. Among these, spilanthol is the most abundant and has demonstrated notable insecticidal efficacy due to its strong ability to penetrate insect tissues and disrupt the central nervous system [39,48].

The insecticidal activity of extracts from *A. oleracea* against a relatively wide range of insect species has been investigated, demonstrating high toxicity, particularly against larvae of mosquitoes, belonging to the family Culicidae (Diptera), such as *Anopheles culicifacies* Giles, *Anopheles stephensi* Liston, *Culex quinquefasciatus* Say, and *Aedes aegypti* (Linnaeus) [49,50,51,52]. Significant larvicidal effects have also been reported against larvae of the moths *T. absoluta*, *Plutella xylostella* (Linnaeus) (Lepidoptera: Plutellidae), and *Spodoptera littoralis* (Boisduval) (Lepidoptera: Noctuidae) [51,53,54]. In addition, the maize weevil *Sitophilus zeamais* (Motschulsky) (Coleoptera: Curculionidae), the housefly *Musca domestica* L. (Diptera: Muscidae), the aphids *Myzus persicae* (Sulzer) and *Lipaphis erysimi* (Kaltenbach) (Hemiptera: Aphididae), and the American cockroach *Periplaneta americana* (L.) (Dictyoptera: Blattidae) have shown susceptibility to the plant’s bioactive compounds [51,55,56,57]. Furthermore, its acaricidal potential has been evaluated against the tomato russet mite *Aculops lycopersici* (Massee) (Acari: Eriophyidae) and the ticks *Rhipicephalus microplus* (Canestrini) and *Dermacentor nitens* (Neumann) (Ixodidae) [58,59,60,61]. Although few studies have addressed the side effects of *A. oleracea* extracts or single compounds towards useful arthropods, no negative effects were found on the aphid parasitoid *Diaeretiella rapae* McIntosh (Hymenoptera: Braconidae) and the aphid predator *Orius insidiosus* (Say), (Hemiptera: Anthocoridae) [44]. Additionally, three *N*-alkylamides occurring in the *A. oleracea* extract proved to be selective towards *Solenopsis saevissima* (Smith) (Hymenoptera: Formicidae), a predator of *T. absoluta* (Meyrick) (Lepidoptera: Gelechiidae) and the pollinator *Tetragonisca angustula* (Latr.) (Hymenoptera: Apidae: Meliponinae) [40]. Moreover, *A. oleracea* compounds exhibited limited toxicity toward non-target beneficial aquatic species [62,63]. More recently, an *A. oleracea* extract enriched in *N*-alkylamides showed a reduced effect towards the mite predator *Typhlodromus exhilaratus* Ragusa (Acari: Phytoseiidae) [61].

However, as in almost all studies *A. oleracea* or single compounds were not applied in NEs formulations, in the present study, we developed NEs containing different amounts of *N*-alkylamides enriched-fraction (AEF) extracted from *A. oleracea* through green technologies, namely supercritical CO_2_ extraction combined with wiped-film short path molecular distillation. The NEs’ activity against eggs, larvae, and adults of *T. absoluta* was evaluated in laboratory experiments.

## 2. Materials and Methods

### 2.1. Chemicals and Reagents

High Performance Liquid Chromatography (HPLC)-grade acetonitrile and methanol employed for HPLC analyses were acquired from Carlo Erba Reagents s.r.l. (Milan, Italy). Spilanthol, used as the reference standard for the HPLC-photoDiode Array Detector (DAD) and Mass Spectrometer (MS) analysis, was isolated by subsequent flash and gravity chromatography and by employing a gradient solvent system as reported by Ferrati et al. (2024) [64]. The structure and purity of the compound were confirmed by ^1^H and ^13^C NMR (Nuclear Magnetic Resonance), and they were in accordance to that previously reported [39].

### 2.2. Acmella oleracea Plant Material

*Acmella oleracea* flowering aerial parts were obtained from Dr. Ettore Drenaggi’s plantation (Castelfidardo, Italy, 43°27′16″ N; 13°31′52″ E, 38 m a.s.l.) by manual harvesting in August 2023. The cultivation was carried out as reported in the work of Ferrati et al. (2024) [64]. After drying, the plant material was shredded to 1.5 mm particle size, employing a plant shredder (Albrigi, mod. E0585, Stallavena, Verona, Italy).

### 2.3. AEF Preparation

The AEF was obtained by subsequently combining two green technologies, namely supercritical CO_2_ extraction and wiped-film short path molecular distillation (WSMD). Firstly, dry aerial parts of *A. oleracea* were extracted with supercritical CO_2_ by using a TH22–10 ×2 supercritical CO_2_ extraction equipment purchased from Toption Instrument Co., Ltd. (YanTa District, Xi’an, China). The extract (3.85% yield) was then fractionated by WSMD with a VKL 70–5 FDRR-SKR-T short path distillator purchased from VTA Verfahrenstechnische Anlagen GmbH & Co. KG (Niederwinkling, Germany) to obtain the AEF (35% yield). Details on both the extraction and fractionation procedures are reported in the work of Ferrati et al. (2024) [64].

### 2.4. HPLC-DAD-MS Analysis

#### 2.4.1. Samples and Standard Solutions Preparation

Stock solutions at 1000, 500, and 100 ppm of spilanthol were prepared in MeOH and kept at −20 °C until use. Additional solutions were prepared by diluting the initial stock solutions to 250, 50, 10, 5, and 1 ppm, respectively. In contrast, the AEF was solubilized at 1000 ppm in acetonitrile by vortex and ultrasounds (Analogic ultrasonic bath Mod. AU-220, ARGOLAB, Carpi, Italy). All solutions were filtered through a 0.2 μm syringeless filter before being analysed.

#### 2.4.2. Analytical Conditions and Method Validation

The HPLC-DAD-MS analysis was conducted using an HPLC instrument Agilent 1100 series (Agilent Technologies, Santa Clara, CA, USA), with a DAD. It also comprised an autosampler, a binary solvent pump, and an ion-trap mass spectrometer (with electrospray ion source set in the positive mode) LC/MSD Trap SL Agilent Technologies. The analytes were separated by employing a Luna C18 column (4.6 × 150 mm, i.d., particle size 5 μm), purchased from Phenomenex (Chesire, UK). The analytical parameters were linear with those previously developed by Kavallieratos et al. (2023) [41]. Spilanthol and the minor *N*-alkylamides were quantified through the DAD detection system at 220 nm, while their structure was confirmed through MS analysis in accordance with Kavallieratos et al. (2023) [41]. The method was validated in terms of linearity (Figure S1, Supplementary material). The linear regression equation found for spilanthol calibration curve was y = 7.6971x + 23.423, and the coefficient of determination (R^2^) was 0.9995 (Figure S1, Supplementary Material). The method was also validated in terms of repeatability, limits of detection (LODs), limits of quantification (LOQs), linearity, and precision. The method repeatability was examined in terms of the Relative Standard Deviation (RSD)%, injecting the 250 ppm standard solution 3 times on the same day (intraday) (0.27%) and 3 times in 3 consecutive days (interday) (0.53%). The LOD (0.6 ppm) was calculated with a signal-to-noise ratio (S/N) of 3:1 and the LOQ (1.75 ppm) with an S/N of 10:1.

### 2.5. Preparation and Characterization of NEs

The NEs were prepared by a high-energy method employing ultrasounds. In detail, the corresponding amount of AEF to reach the final concentrations of 0.06, 0.125, 0.25, and 0.5% (*w*/*w*) in the NEs was mixed with ethyl oleate (1% *w*/*w* of the NEs). Then, the oil phase was added dropwise to a polysorbate 80 (2% *w*/*w* of the NEs) aqueous solution. The two phases were vigorously mixed under high-speed stirring (Ultraturrax T25 basic, IKA^®^ Werke GmbH and Co. KG, Staufen, Germany) for 5 min at 13,500 rpm. The obtained emulsions were subjected to ultrasounds by employing a 2L-Ultrasound Extractor U2020 (170 W, 230 V, 50 Hz) (Albrigi Luigi Srl, Verona, Italy) for 40 min using the H + M (high power + homogenization) program to reduce the droplet size. Moreover, a control NE not containing the AEF was also prepared, as previously described.

The NEs were characterized in terms of particle size by a Dynamic Light Scattering (DLS) analysis employing a Zetasizer nanoS (Malvern Instrument, Malvern, UK) following the procedure by Benelli et al. (2020) [34]. The stability of NEs, stored at 4 °C, was determined by analyzing their mean droplet size (Z-average) and polydispersity index (PDI) at different time points: 0 (T0), 15 (T1), 30 (T2), 90 (T3), 180 (T4), and 240 (T5) days.

### 2.6. Tomato Plant Rearing

Plants of tomato for all the experiments were cultivated at the CREA—Research Centre for Plant Protection and Certification (CREA-DC) of Palermo (Italy). Seeds of ‘*San Marzano nano*’ cultivar (ITALSEMENTI^®^ snc.; Arezzo, Italy) were sown in pots (10 × 10 × 15 cm) with 1 L of topsoil enriched peat (Gramoflor^®^, GmbH & Co. KG) added with about 30% of expanded vermiculite (VIC Italiana^®^ s.p.a.; Milan, Italy). Pots were kept in fine mesh (44 × 32 mesh) net cages (45 × 45 × 90 cm) to prevent other undesirable insect species and maintained at 27 ± 2 °C temperature, 50 ± 10% relative humidity (RH), and a photoperiod (L:D) of 14:10 h. Fertilization with Best 20-20-20 (Aifar^®^ s.p.a.; Genova, Italy) was done one time per week. Plants of about 50 cm height and with at least five true leaves were used for experiments and *T. absoluta* rearing.

### 2.7. Insect Laboratory Rearing

*Tuta absoluta* rearing was maintained at the CREA-DC of Palermo. It started with insects collected on infested cultivated tomato plants in eastern Sicily, kindly provided by the Department of Agriculture, Food and Environment (Di3A) of the University of Catania. To reduce the genetic drift, *T. absoluta* individuals from field infested plants were added every 6 months to the rearing cages.

To obtain coetaneous *T. absoluta* instars for the experiments, about 200 adults obtained from the main colony were placed in a fine mesh (44 × 32 mesh) net cage (45 × 45 × 45 cm) containing three tomato plants, removing them after three days. Approximately two weeks later, when the 4th instar larvae became pupae, all the vegetal material was cut and put in another screened cage until the emergence of the coetaneous adults for the experiments. Moreover, once a week, 200 newly emerged adults of *T. absoluta* (24–48 h old) and three tomato plants were put in a screened cage (45 × 45 × 45 cm) with a fine mesh (44 × 32 mesh) and removed after three days, as described above, to obtain the 2nd instar larvae.

The *T. absoluta* rearing and all the following bioassays were carried out in a climatic chamber at 25 ± 2 °C, 50 ± 10% RH, and 16:8 h L:D.

### 2.8. Phytotoxicity Assessment

Two different AEF-NEs containing 0.25 and 0.5% (*w*/*w*) of AEF a.i., respectively, were used to assess the phytotoxicity. The two AEF-NEs were compared with a control NE solution (i.e., the AEF-free NE) and a negative control (distilled water). Treatments on leaves were applied using a hand sprayer, until the solution ran off. Each treatment was applied on 5 tomato plants. The treated plants were then placed in a greenhouse and checked 1, 3, 7, and 14 days after the treatment. The percentage of damaged leaves was recorded, and the damage severity was assessed following the classification used by Campolo et al. (2017) [12]: (i) 0, no damage; (ii) 1, leaf surface partially damaged with chlorosis but without necrosis; (iii) 2, leaves with evident necrosis; (iv) 3, dead leaves. Moreover, the phytotoxicity index (*Pi*) was calculated as follows:

$$Pi=\sum_{j=0}^{n}\left(\frac{{DL}_{j}}{TL}\times \frac{DC}{n-1}\right)$$

in which DL is the number of damaged leaves for each damage severity class j; TL is the total number of sprayed leaves; DC is the damage severity class; n is the number of damage severity classes.

*Pi* ranges from 0 (no damage) to 1 (dead leaves).

### 2.9. Topical Toxicity on Eggs

Based on the results obtained in phytotoxicity trials, we tested the ovicidal effect of AEF-NE at 0.25, 0.125, and 0.06% a.i. in comparison with a negative control (distilled water) and a control NE solution (the AEF-free NE), following the protocol described by Tortorici et al. [65]. Untreated tomato sprouts and 100 mated adults of *T. absoluta* (about 72 h old) were put in a cage (45 × 30 × 45 cm) with a fine mesh (44 × 32 mesh) net for 24 h and then removed. After 24 h from insect removal, the sprouts were picked up from the cage, sprayed until dripping and let dry for 60 min. After drying, the eggs were moved with a fine brush from the treated sprouts to untreated tomato leaves, which were placed in a two-cup experimental arena as described by Biondi et al. (2012) [66]. For each treatment 5 replicates were performed, each consisting of 10 treated eggs per leaf (50 eggs per treatment). The eggs were daily checked for 7 days, recording their hatching.

### 2.10. AEF-NE Activity on T. absoluta Larvae: Topical Toxicity

To evaluate the topical toxicity of AEF-NEs against *T. absoluta* larvae, treatments were carried out with the same three concentrations used in the egg bioassay (0.25, 0.125 and 0.06% a.i.). A negative control (distilled water) and a control NE (NE without AEF) were tested. The 2nd instar larvae used in the trials were taken from insect rearing, put on absorbent paper sheet, sprayed with 15 mL of each solution and let dry for 15 min. For each treatment, 6 replicates were performed with 5 treated larvae (2.5 mL of solution), for a total of 30 treated larvae per treatment. The larvae were then removed with a fine brush and placed on untreated tomato leaves in the two-cup experimental arena, as described above. The larvae were checked at 24, 48, and 72 h after treatment, recording their survival. Furthermore, 15 days after treatment, the emerged adults were counted.

### 2.11. AEF-NE Activity on T. absoluta Larvae: Ingestion Toxicity

The ingestion toxicity against *T. absoluta* 2nd instar larvae was tested at 0.25, 0.125, and 0.06% a.i. concentrations. Both the negative control and control NE were tested as well. Untreated tomato leaves were sprayed as described above and dried in laboratory conditions for 60 min. The treated leaves were stored in the two-cup experimental arena at the same laboratory conditions of the trials carried out on eggs. Afterwards, the 2nd instar larvae taken from insect rearing were moved with a fine brush on treated leaves. A total of 30 *T. absoluta* larvae per treatment were used (6 replicates, each consisting of 5 larvae). Larvae were checked at 24, 48, and 72 h after treatment, recording their survival. Finally, 15 days after treatment, the emerged adults were counted.

### 2.12. Ovideterrence Experiments

The repellent activity of the AEF-NEs was evaluated in two-choice assays at the concentrations of 0.25 and 0.125% a.i. on *T. absoluta* egg laying females, following the experimental protocol by Tortorici et al. (2025) [65]. Five different comparisons were tested: (i) distilled water vs. control NE, (ii) distilled water vs. AEF-NE (0.125%), (iii) distilled water vs. AEF-NE (0.25%), (iv) AEF-NE (0.125%), vs. control NE, (v) AEF-NE (0.25%), vs. control NE. Tomato leaves were sprayed (see “Phytotoxicity assessment” paragraph) and let dry for 60 min in laboratory conditions. Subsequently two treated leaves, each put in a tube with distilled water, were placed in a plastic box (45 × 30 × 45 cm) with two aerated holes covered with a fine mesh net (44 × 32 mesh), following the comparisons reported above. For the experiment, 72 h old adults were kept in a cage in which both sexes were present to allow adult mating. In each experimental cage, 15 adults (10 female and 5 male) were released for oviposition. After 48 h, adults were removed, and the total number of laid eggs on each tomato leaf was counted. Three replicates for each two-choice assay were performed (30 females and 15 males in total).

### 2.13. Data Analysis

Data from the toxicity and ovideterrence trials were checked through the Levene test (*p* > 0.01) for homogeneity and the normality of the dependent variables whenever needed. Data were evaluated through GLM Univariate analysis for multiple mean comparisons among treatments (*p* ≤ 0.05), followed by Tukey’s HSD post hoc tests. The phytotoxicity index (*Pi*) was analysed with GLM Univariate followed by Tukey’s HSD post hoc tests, with concentrations and time after the treatment as fixed factors and *Pi* as the dependent variable. Statistical analyses were carried out using SPSS 25.0 software (IBM Corp., Armonk, NY, USA).

## 3. Results

### 3.1. HPLC-DAD-MS Analysis of AEF

The identification of the main *N*-alkylamides in AEF was performed according to Kavallieratos et al. (2023) [41], and their concentration, expressed as g/100 g of extract, is reported in Table 1.

The total *N*-alkylamides content was 46.22 g/100 g, and the main representative of this class was confirmed to be spilanthol, with a concentration of 42.40 g/100 g. Other *N*-alkylamides were (2*E*,6*Z*,8*E*)-*N*-(2-methylbutyl)-2,6,8-decatrienamide (2.71 g/100 g), (2*Z*)-*N*-isobutyl-2-nonene-6,8-diynamide (0.66 g/100 g), (2*E*)-*N*-isobutyl-2-undecene-8,10-diynamide (0.45 g/100 g), and (2*E*,7*Z*)-*N*-isobutyl-2,7-decadienamide, and (2*E*)-*N*-(2-methylbutyl)-2-undecene-8,10-diynamide (0.50 g/100 g).

### 3.2. Preparation and Characterization of NEs

**Figure 1:**
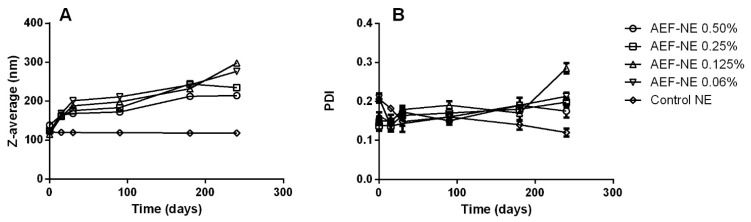
Variation in Z-average (nm, (**A**)) and polydispersity index (PDI, (**B**)) for AEF-NEs at different concentrations (0.5, 0.25, 0.125, and 0.06% a.i. *w*/*w*) and control NE over time (up to 240 days, stored at 4 °C).

DLS was employed to assess the formation of nanosized oil droplets in AEF and control NEs, as well as to monitor their physical stability over time. The mean droplet size, expressed as the Z-average value, and the grade of polydispersity, expressed as the polydispersity index (PDI), are reported in Figure 1A and Figure 1B, respectively. After the preparation, all formulations exhibited a Z-average in the range of 110–140 nm and a PDI between 0.140 and 0.210, confirming the successful formation of nanoemulsified systems with narrow droplet size distributions. The physical stability was monitored by following the variations of Z-average and PDI during an observation time of 240 days. The control NE maintained nearly constant Z-average and PDI values throughout the storage period, demonstrating excellent physical stability in terms of droplet size distribution. In contrast, the AEF-loaded NEs showed a modest increase in Z-average (mainly occurring in the first 30 days), reaching values between 220 and 290 nm at the end of the observation time, while the PDI values remained around 0.2. Despite this slight size increase, the overall results support the effective formation and long-term physical stability of the AEF-NEs, indicating their suitability for practical applications for at least eight months from their preparation.

### 3.3. Phytotoxicity Assessment

**Figure 2:**
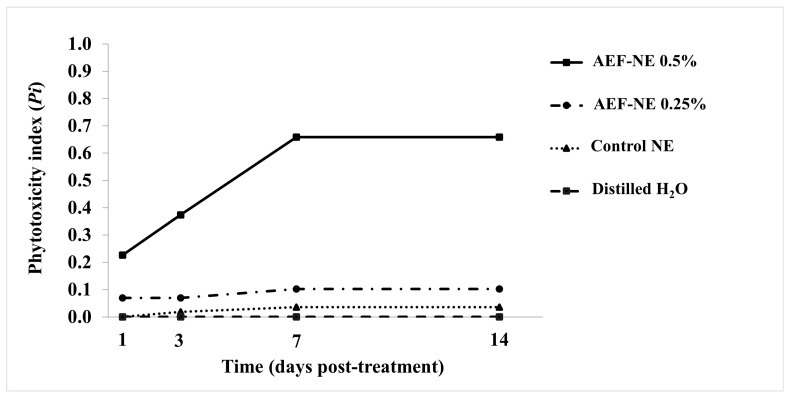
Phytotoxicity index (*Pi*, sum values) of AEF-NE 0.25, and 0.5% a.i., control NE and distilled water recorded at 1, 3, 7, and 14 days post-treatment on tomato plants (5 plants per treatment). (GLM univariate followed by Tukey’s HSD post hoc, *p* < 0.05).

The results of the test carried out to assess the toxic effect of the different concentrations of AEF-NEs on plants are reported in Figure 2. At each observation date, the highest phytotoxicity index (*Pi*) was recorded in the treatment with AEF-NE 0.5% a.i., with *Pi* = 0.658 after 14 days. In treatments with AEF-NE 0.25% and control NE, slight phytotoxicity was recorded after 14 days (*Pi* = 0.102 and *Pi* = 0.036 respectively), while in the treatment with distilled water no phytotoxicity was recorded (*Pi* = 0). The statistical analysis showed that the *Pi* significantly depends on the treatment (*F_3,16_* = 145.043; *p* = 0.000) and on the days after the treatment (*F_3,16_* = 11.580; *p* = 0.000). Moreover, in all days post-treatment, the phytotoxicity in AEF-NE 0.5% was significantly higher than all the other treatments, while no significant differences were found among them (GLM univariate performed for each day: day1: *F_3,16_* = 16.404, *p =* 0.001; day3: *F_3,16_* = 22.081, *p* = 0.000; day 7: *F_3,16_* = 53.882, *p* = 0.000; day 14: *F_3,16_* = 53.882, *p* = 0.000).

### 3.4. Topical Toxicity on Eggs

**Figure 3:**
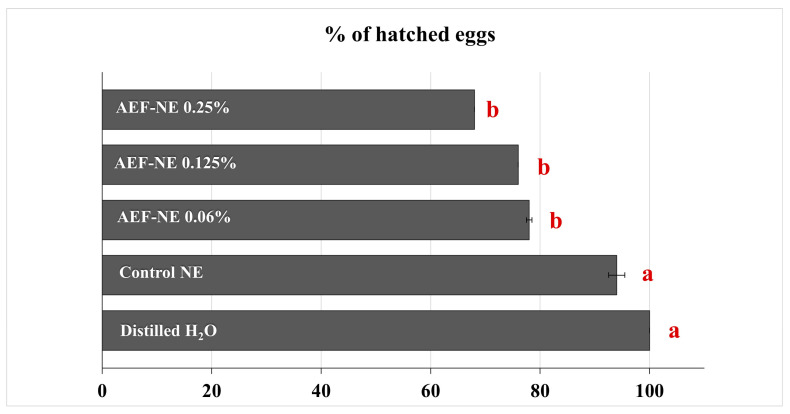
Percentage of *Tuta absoluta* hatched eggs (n = 50 eggs per treatment) after treatment with AEF-NE 0.06, 0.125, 0.25% a.i., control NE, and distilled water. Horizontal bars indicate SE. Different letters indicate significant differences at *p* < 0.05 (GLM univariate, Tukey’s HSD post hoc).

The highest percentage of hatched eggs was recorded in the distilled water (100.0%) and in the control NE (94.0%), followed by AEF-NE 0.06% a.i. (78.0%), AEF-NE 0.125% a.i. (76.0%), and AEF-NE 0.25% a.i. (68%) (Figure 3). The percentage of hatched eggs did not significantly differ among the three AEF-NE concentrations but was significantly lower than the two control treatments (*F_4,25_* = 29.533, *p* = 0.000).

### 3.5. Topical Toxicity on Larvae

**Figure 4:**
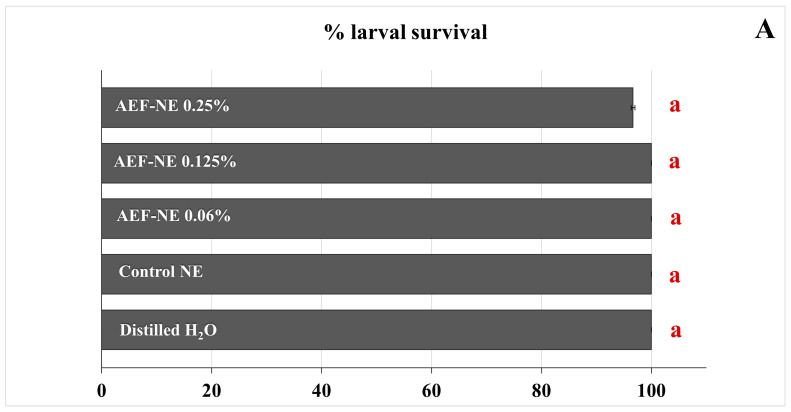

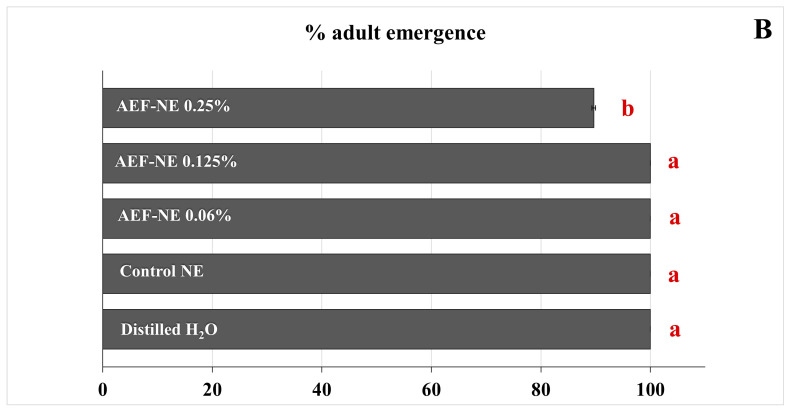
Percentage of *Tuta absoluta* larval survival (n = 30 larvae per treatment) after 72 h (**A**) and of adult emergence (**B**) in topical toxicity bioassays testing AEF NE 0.125, 0.25% a.i., control NE, and distilled water. Horizontal bars indicate SE. Different letters indicate significant differences at *p* < 0.05 (GLM univariate, Tukey’s HSD post hoc).

The percentage of larval survival was high and not significantly different in all treatments, ranging from 96.7% in AEF-NE 0.25% a.i. to 100% in all other treatments immediately after 24 h (*F_4,25_* = 1.000, *p* = 0.426) (Figure 4A). However, the larvae that survived the treatment AEF-NE 0.25% a.i. showed a percentage of adult emergence (89.7%) significantly lower than all the other treatments (100%) (*F_4,25_* = 10.000, *p* = 0.000) (Figure 4B).

### 3.6. Ingestion Toxicity on Larvae

**Figure 5:**
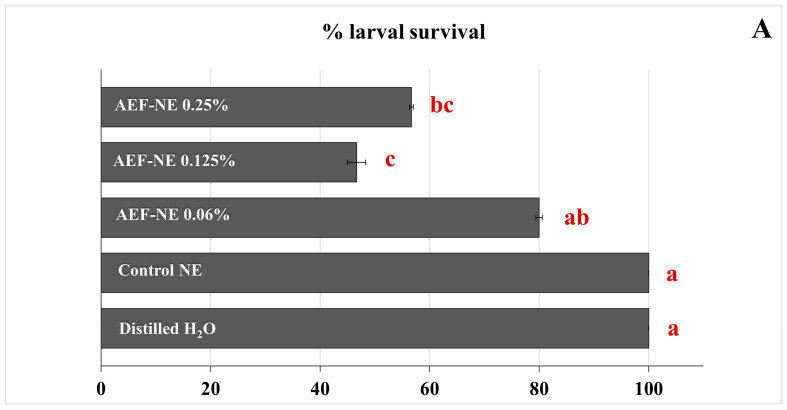

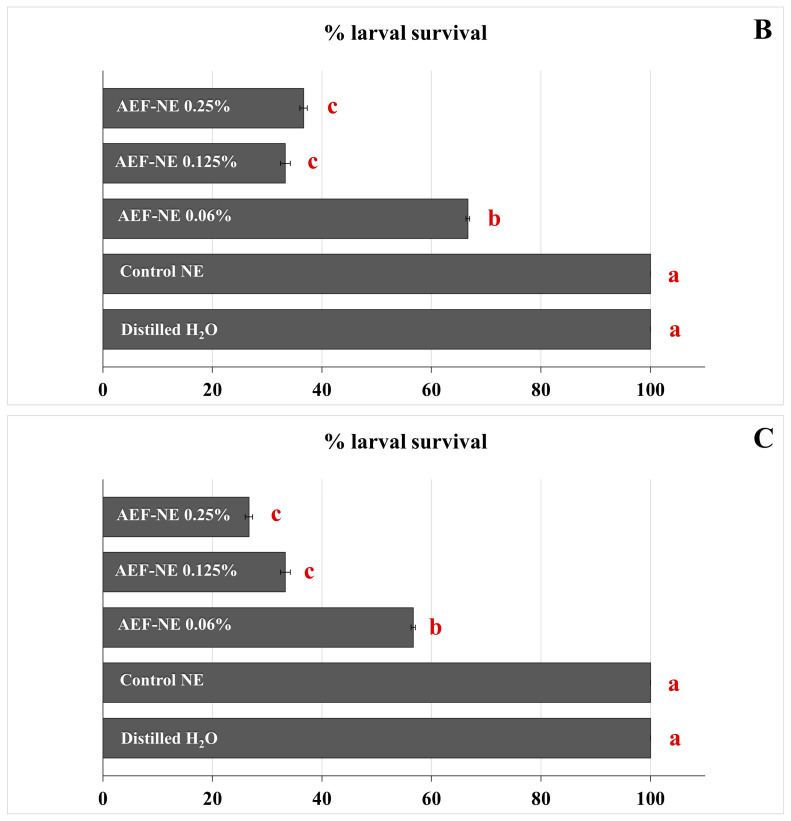

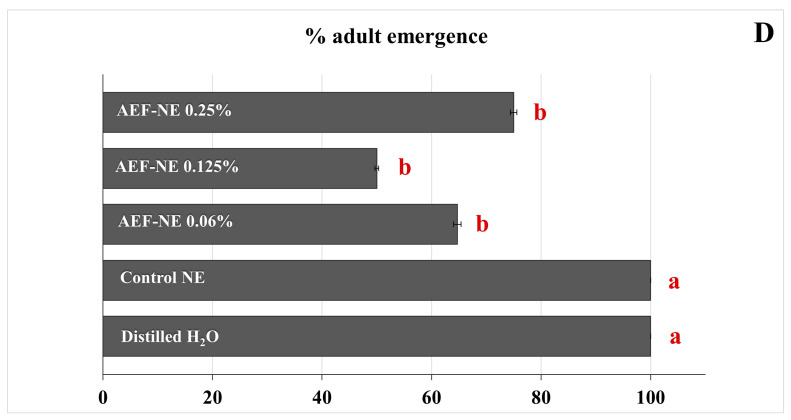
Percentage of *Tuta absoluta* larval survival (30 larvae per treatment) after 24 h (**A**), 48 h (**B**) and 72 h (**C**) and of adult emergence (**D**) in ingestion toxicity bioassays testing AEF NE 0.125, 0.25% a.i., control NE, and distilled water. Horizontal bars indicate SE. Different letters indicate significant differences at *p* < 0.05 (GLM univariate, Tukey’s HSD post hoc).

All three AEF-NE concentrations used in the trials, 0.06, 0.125 and 0.25% a.i., showed a significant toxic effect after 24 h (*F_4,25_* = 15.882, *p* = 0.000), 48 h (*F_4,25_* = 41.754, *p* = 0.000) and 72 h (*F_4,25_* = 40.580, *p* = 0.000). Significant differences in the larval survival percentage were found between the two 0.125 and 0.25% AEF-NEs concentrations and the 0.06% one (Figure 5A). Moreover, after 15 days, the percentage of adult emergence from larvae treated with the different AEF-NEs concentrations was significantly lower than distilled water and the control NE (*F_4,25_* = 68.664, *p* = 0.000) (Figure 5B).

### 3.7. Ovideterrence Activity

**Figure 6:**
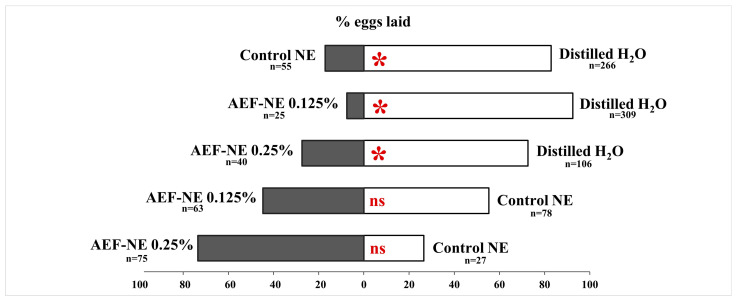
Percentage of *Tuta absoluta* eggs laid on tomato sprouts in ovideterrence two-choice assays. Distilled water vs. control NE; distilled water vs. AEF-NE 0.125%; control NE vs. AEF-NE 0.125%; distilled water vs. AEF-NE 0.25%; control NE vs. AEF-NE 0.25%. n = number of laid eggs; * = significant, ns = not significant (GLM univariate, Tukey’s HSD post hoc, *p* < 0.05).

In the two-choice experiments, the percentage of eggs laid on tomato sprouts treated with distilled water was always significantly higher compared with control NE (*F_1,4_* = 47.769, *p* = 0.002), AEF-NE 0.125% a.i. (*F_1,4_* = 44.710, *p* = 0.003) and AEF-NE 0.25% a.i. (*F_1,4_* = 17.780, *p* = 0.014). No differences were found in the two tests in which the control NE was compared to AEF-NE 0.125% a.i. (*F_1,4_* = 0.262, *p* = 0.636) and AEF-NE 0.25% a.i. (*F_1,4_* = 5.606, *p* = 0.077) (Figure 6).

## 4. Discussion

*Acmella oleracea* is an extensively cultivated plant worldwide that is widely used in food, cosmetics, pharmaceuticals, and agricultural science [39]. Its multiple properties are mainly due to *N*-alkylamides, specifically spilanthol [67]. The chemical analysis of AEF was consistent with our previous work [64]. The main *N*-alkylamide detected was spilanthol, although its concentration in the extract was slightly lower (42.4 g/100 g) than that previously reported (55.9 g/100 g) [64]. This is probably ascribed to the different *A. oleracea* batch employed for the AEF production. Nevertheless, our AEF was richer in *N*-alkylamides than other *A. oleracea* extracts obtained with other procedures [41,68,69,70,71].

The high-energy method employing ultrasounds proved to be highly effective for the nanoemulsification of AEF from *A. oleracea*, leading to the formation of NEs with nanosized droplets and narrow size distributions, as evaluated in terms of the Z-average and PDI. These findings reinforce the suitability and versatility of ultrasound-assisted methodologies for the encapsulation of plant-derived extracts, thereby achieving disperse phases within the nanometric range. Notably, the Z-average and PDI values obtained for the AEF-NEs were comparable to those reported in previous studies employing the same technique to encapsulate *N*-alkylamides from *A. oleracea* hexane extract [72] and carlina oxide isolated through hydrodistillation of *Carlina acaulis* L. (Asteraceae) roots [20,65].

Recent studies showed that *A. oleracea* extract can also be an interesting alternative to the use of synthetic products for the control of arthropod pests [41,53]. In our experiments with AEF-NE, although the concentrations of a.i. used were less than 0.5% due to the phytotoxicity on tomato plants, the results on *T. absoluta* seem to be promising. The phytotoxicity damage could certainly be reduced with the use of other nanotechnologies (e.g., carrier formulations). These latter, when combined with NEs, could further reduce the phytotoxic effect. However, the use of reduced doses of the active ingredient allowed by nanotechnologies is a positive aspect also from the perspective of a lower production cost. Regarding the results, a significant difference was obtained in terms of the percentage of hatched eggs and of larvae surviving ingestion in the toxicity tests between the AEF-NEs and the relative controls.

Although some *N*-alkylamides have shown clear biological activity [39], the mechanism of action on arthropods and on vertebrates is almost unknown and will require further specific investigations. Different concentrations of *A. oleracea* extract have been demonstrated to cause alterations in the ovaries and midgut cells of semi-engorged females of the thick *Rhipicephalus sanguineus* Latreille, 1806 (Acari: Ixodidae) [73]. Spilanthol has been supposed to act on the central nervous system of the insect, causing abnormal movements such as jerks, spinning, and uncoordinated muscular activity [53,74]. Spilanthol and other alkylamides have been investigated also for their anesthetic or antipyretic activity on humans, leading to the hypothesis that they can influence GABA release or sodium channels [75,76,77,78].

Our topical tests on larvae did not show significant differences between AEF-NEs and the controls. Moreno et al. (2012) [53] found that the hexane extract from aerial parts of *A. oleracea* dissolved in acetone showed good insecticidal activity against *T. absoluta*. The different results may be due to the completely different products. Firstly, the *N*-alkylamides fraction is a fraction enriched in *N*-alkylamides (around 50% of the total composition), and this is not the case of the hexane extract, whose chemical composition was not reported. So, it could be possible that other lipophilic compounds played a role in the bioactivity, maybe enhancing the penetration of the bioactive compounds. Moreover, it could be possible that the application mode influenced the activity. Indeed, Moreno et al. (2012) [53] tested the extract or the pure compounds directly dissolved in acetone. Differently, we encapsulated the compounds into NEs, which probably needed a longer exposure time to exert their bioactivity. Moreover, the different results could be explained by the synergistic or antagonistic effect of all the extracted components, but the harvesting period, the portion of the plant used, the solvent, and the extraction methodology could also play a role [41,52,54].

On the other hand, the results obtained in the ingestion tests showed a significant dose-dependent mortality of all the AEF-NEs concentrations. Nevertheless, in both the topical and ingestion experiments, the AEF-NEs affected the growth of the surviving treated larvae and their metamorphosis, reducing the adult emergence. This effect was more evident in the ingestion trial than in the topical one. In general, *N*-alkylamides are able to affect the development and reproduction of the treated insects, including pupation and adult emergence [79]. Saraf and Dixit (2002) [74] described a high pupal mortality of *C. quinquefasciatus*, *An. culicifacies* and *Ae. aegypti* after spilanthol treatment.

Lastly, unlike the toxicity tests, where the AFN-NEs showed in almost all cases a significantly higher activity compared to the control NE, in the two choice tests, a significant oviposition deterrence effect was recorded for both the control NE and AFN-NEs compared with distilled water but not for AEF-NEs compared with the control NE. Tortorici et al. (2025) [65] found similar results when testing carlina oxide NEs at different concentrations, suggesting that the repellent activity may depend on the ethyl oleate present in NE. Few studies are currently available on the effect of ethyl oleate, although it is a component of extracts from different plant species, where it can be present with variable concentrations [80,81,82]. Ethyl oleate has been tested against the stored pest *Callosobruchus maculatus* (Fabr.) (Coleoptera: Bruchidae), negatively affecting the oviposition period and reducing the number of eggs laid in comparison to the control [83]. Moreover, on *S. zeamais*, it caused a significant reduction in the number of laid eggs, whereas two-choice repellency test oviposition showed a low repellent effect, not significantly different compared to the control [80].

Overall, considering the simultaneous toxicity on eggs and larvae and the ovideterrence on adult females, the AEF formulated in NEs could be considered as a potential alternative to common insecticides against *T. absoluta*. Furthermore, given the resistance to insecticides by *T. absoluta*, the rotation of naturally occurring insecticides in integrated pest management programs may be a promising alternative. Among other things, these NEs would also be easily applicable in the field with the common spraying machines already used by farmers. However, further studies are required to fill the knowledge gap on the potential use of this product as an ecofriendly insecticide, such as the assessment of its mode of action (i.e., penetration, effects on the insect tissues), the stability and effectiveness under field conditions, and the bioactivity on non-target species, especially biological control agents and pollinators.

## Figures and Tables

**Table 1:** *N*-alkylamides content in AEF determined by HPLC-DAD-MS analysis.

	*N*-Alkylamide	Concentration (g/100 g) ^a^ ± SD ^b^	RSD% ^c^
1	(2*Z*)-*N*-isobutyl-2-nonene-6,8-diynamide	0.66 ± 0.00	0.69
2	(2*E*)-*N*-isobutyl-2-undecene-8,10-diynamide	0.45 ± 0.00	0.39
3	(2*E*,6*Z*,8*E*)-*N*-isobutyl-2,6,8-decatrienamide (spilanthol)	42.40 ± 0.10	0.24
4	(2*E*,7*Z*)-*N*-isobutyl-2,7-decadienamide	0.50 ± 0.02	3.66
5	(2*E*)-*N*-(2-methylbutyl)-2-undecene-8,10-diynamide		
6	(2*E*,6*Z*,8*E*)-*N*-(2-methylbutyl)-2,6,8-decatrienamide	2.71 ± 0.01	0.38
	Total	46.22 ± 0.13	

^a^ Concentration (g/100 g) represents the average concentration of *N*-alkylamides found in the *N*-alkylamides-enriched fraction (AEF), and it is the mean of three independent analyses; ^b^ SD = standard deviation; ^c^ RSD% = relative SD.

## Data Availability

The original contributions presented in this study are included in the article. Further inquiries can be directed to the corresponding authors.

## Supplementary Materials

The following supporting information can be downloaded at: https://www.mdpi.com/article/10.3390/insects17050455/s1, Figure S1: Spilanthol calibration curve employed for HPLC-DAD-MS analysis.
