# Supplementary figures and images for: Encapsulation of a N-Alkylamide-Enriched Fraction from Acmella oleracea and Its Efficacy Against Tuta absoluta, the Invasive Key Tomato Pest
Source: Insects. 2026 Apr 26;17(5):455. doi: 10.3390/insects17050455 (PMC13206765; doi:10.3390/insects17050455)

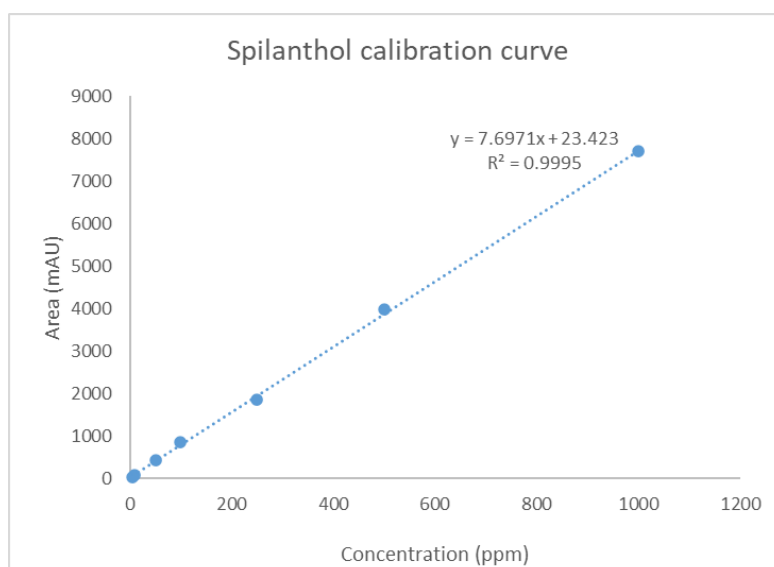

**Figure S1.** Spilanthol calibration curve employed for HPLC-DAD-MS analysis.

Supplement: Supplementary file 1 [file insects-17-00455-s001.zip › insects-4221973-supplementary.pdf]
